# Social defeat stress induces liver injury by modulating endoplasmic reticulum stress in C57BL/6J mice

**DOI:** 10.1038/s41598-024-57270-0

**Published:** 2024-03-26

**Authors:** XiaoLei Gao, Tong Zhao, Ran Hao, ZhaoHui Zhang, Guang-Biao Huang

**Affiliations:** 1 School of Nursing, Xinxiang Medical University, Xinxiang, China; 2 Henan Key Laboratory of Biological Psychiatry, Xinxiang Medical University, Xinxiang, China; 3 Department of Psychiatry, QuZhou Third Municipal Hospital, QuZhou, China; 4 The First Affiliated Hospital of Xinxiang Medical University, Xinxiang, China; 5 Department of Psychiatry, Huzhou Third Municipal Hospital, The Affiliated Hospital of Huzhou University, No. 2088, Tiaoxi East Road, Huzhou, 313000 China

**Keywords:** Apoptosis, Endothelium reticulum stress, Inflammation, Liver injury, Social defeat stress, Anxiety, Depression

## Abstract

Social defeat stress is associated with endoplasmic reticulum (ER) stress, inflammation and apoptosis. ER stress is thought to contribute to many lifestyle diseases such as liver injury, cardiovascular dysfunction and depression. We investigated the expression of the ER stress markers RNA-dependent protein kinase-like ER kinase (PERK), eukaryotic translation initiation factor 2α (eIF2α) and C/EBP homologous protein (CHOP), as well as inflammatory and apoptotic factors, to assess how social defeat stress induces liver injury. Furthermore, we evaluated the effects of the ER stress inhibitor phenylbutyric acid (PBA) and ER stress inducer thapsigargin (TG) on liver injury. Adult mice were divided into the control, social defeat, social defeat + PBA, TG, PBA and TG + PBA groups. The social defeat and social defeat + PBA groups were simultaneously exposed to social defeat stress for 10 days. The social defeat + PBA, TG, PBA and TG + PBA groups were treated with PBA or TG via intraperitoneal injections. PBA was injected 1 h before the TG injection into the TG + PBA group. Liver samples from six groups of mice were analyzed by histological analysis and western blotting. Social defeat stress promoted ER stress, increased the expression of inflammatory factors and induced apoptosis in the liver of socially defeated mice, which was reversed by PBA. Moreover, ER stress induces TG-induced liver injury by initiating ER stress. Social defeat stress initiates ER stress, promotes the expression of inflammatory and apoptotic factors, and induces liver injury. PBA suppresses liver injury caused by social defeat stress and TG treatment.

## Introduction

Stress can be defined as any threat or perceived threat that disturbs an organism’s ability to maintain homeostasis^1^. It is known to accelerate the progression of many disorders^2^. Social defeat stress, as a common social stressor, can increase endoplasmic reticulum (ER) stress marker levels, further inducing inflammation and apoptosis^3^. ER stress is thought to contribute to many lifestyle diseases such as liver injury and depression^4^.

The ER is crucial for the synthesis and folding of proteins, lipid synthesis and calcium storage. ER dysfunction leads to the accumulation of unfolded or misfolded proteins, which in turn accelerate ER stress. The liver is a vital organ that performs metabolic, secretory, and excretory functions. Notably, 70% of liver cells are enriched in ER and are susceptible to ER stress^5,6^. Liver macrophages (Kupffer cells), the key components of hepatic innate immune system, represent the first line of defence in detecting the invading pathogens in the liver. Stress activates macrophages and triggers innate immune responses, and then leading liver inflammation and injury^7^. Numerous studies^3,8^ have indicated that social defeat stress increases the expression of ER stress markers, which have a critical role in the pathogenesis of liver disease. Exposure to restraint or noise stress can induce liver injury, and ER stress is involved in this process^9,10^. However, the exact role of ER stress in social defeat stress-induced liver injury remains unclear.

ER stress triggers the unfolded protein response (UPR), which comprises the IRE1α, PERK and ATF6 signaling pathways and is a protective cellular response activated by ER stress. When ER stress is chronically prolonged and the protein load on the ER greatly exceeds its folding capacity, cellular dysfunction and cell death often occur. Studies have shown that the PERK-eIF2α-CHOP signaling pathway has an important role in liver injury^5,6,11^. CHOP has a convergent role in the UPR; it has been identified as one of the most important mediators of ER stress-induced inflammation and apoptosis. CHOP induces apoptosis via the direct inhibition of Bcl-2 transcription and reduced translation of IκB, thereby increasing NF-κB activity. Elevated expression of NF-κB, TNFα and Bax/Bcl-2 is also involved in acute and chronic liver injury. Moreover, the mechanism of liver injury induced by social defeat stress requires further exploration.

Specifically, PBA is a chemical chaperone that alleviates ER stress in different animal and cell models. Studies have shown that elevated eIF2α phosphorylation can mitigate ER stress during an acute liver injury in mice, and PBA prevents hepatoma cell injury by suppressing ER stress in humans^12,13^. TG is a pharmacological inducer of ER stress that disrupts protein folding in the ER, resulting in ER stress and hepatocyte injury^14,15^. In this study, we used social defeat stress-induced liver injury to investigate the regulatory functions of ER stress-induced inflammation and apoptosis in liver injury. Furthermore, PBA prevented social defeat stress and TG-induced liver injury by initiating ER stress.

## Materials and methods

### Materials

In particular, PBA and TG were purchased from Sigma (Sigma-Aldrich, St. Louis, MO, USA), and all biochemical reagents were purchased from Sigma-Aldrich unless otherwise indicated.

### Animals and treatment protocols

**Figure 1:**
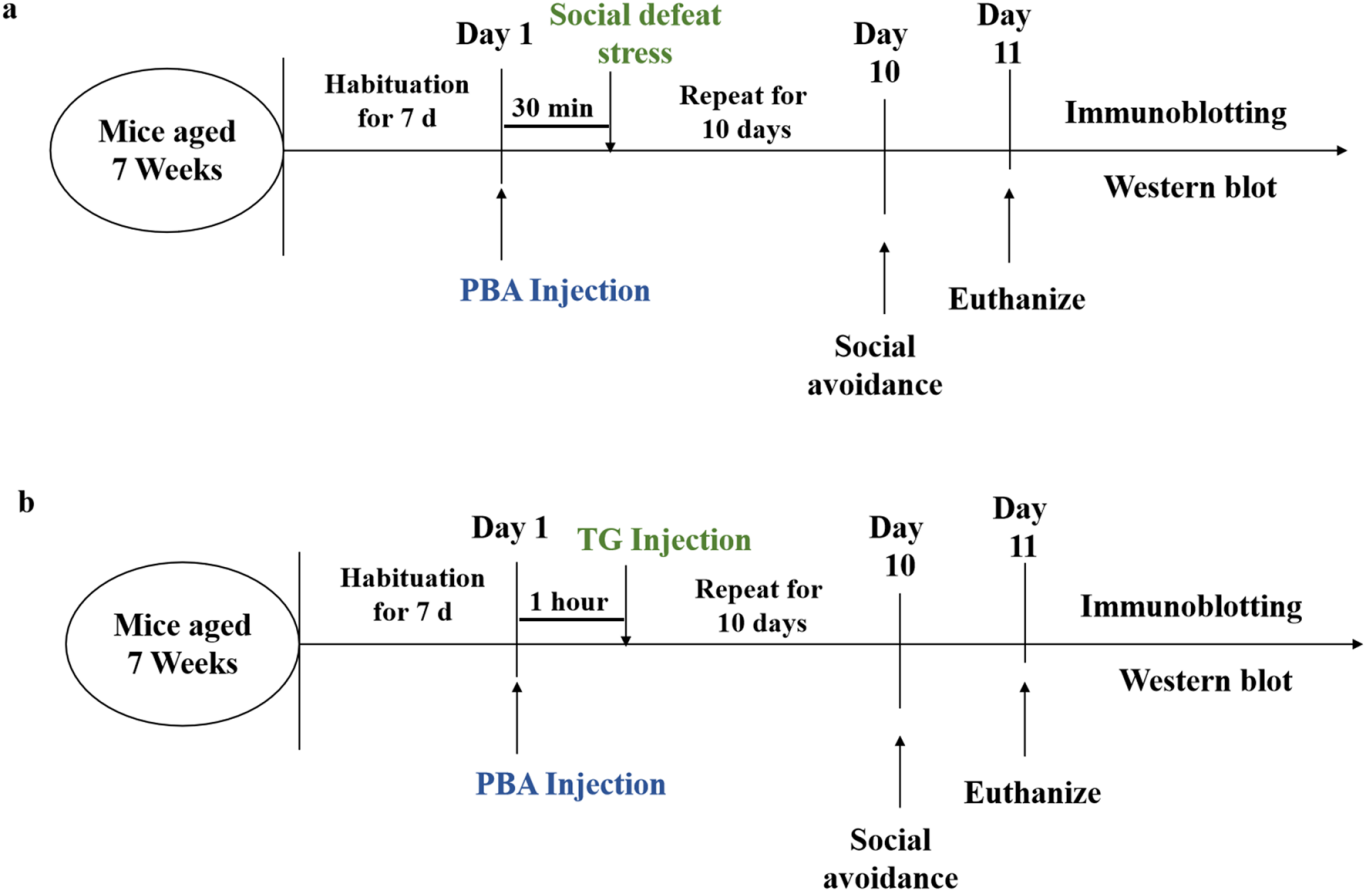
Timeline of experimental procedures. (**a**) Experiment 1: Social defeat group was physically exposed to a different aggressor for 10 min every day for 10 days, Social defeat + PBA group received an intraperitoneal injection of PBA 30 min before social defeat stress. (**b**) Experiment 2: TG group received an intraperitoneal injection of TG every day for 10 days, TG + PBA group received an injection of PBA 1 h before the TG injection. PBA: 4-phenylbutyric acid, TG: Thapsigargin.

Overall, 70 male C57BL/6J mice (7 weeks old, 20–24 g) and 20 male Institute of Cancer Research (ICR) mice (14 weeks old, 40–44 g) were utilized in this study. All mice were housed in groups at the ambient standard room temperature of 22 ± 2 °C, with at least 40% humidity, under a 12-h light/dark cycle (lights on from 07:00–19:00) with food (standard chow diet) and water ad libitum. After one week of acclimatisation, in experiment 1, the C57BL/6J mice were randomly divided into four different experimental groups: (1) control, (2) social defeat, (3) PBA and (4) social defeat + PBA. In experiment 2, the C57BL/6J mice were randomly divided into four different experimental groups: (1) control, (2) PAB, (3) TG and (4) TG + PBA. The social defeat + PBA, PBA and TG + PBA groups received an intraperitoneal injection of PBA (100 mg/kg) according to a previous study^16,17^. In the social defeat and social defeat + PBA groups, all mice were treated once daily during the social defeat stress period. To induce liver injury, the TG and TG + PBA groups were intraperitoneally injected with 2 mg/kg TG according to a previous study^11^. To pharmacologically alleviate ER stress, PBA was injected 1 h before TG injection. The control and social defeat groups were treated with an equal amount of vehicle based on body weight (Fig. 1). After experiments mice were sacrificed by carbon dioxide euthanasia. Dedicated efforts were made to minimize animal suffering and the number of animals used is in accordance with the Guidelines for Animal Experiments of the Xinxiang Medical University, and which was approved by the Ethics Committee of Xinxiang Medical University, China [Date of the permission by Ethics Committees: May 10, 2022; permission number: HMH.No.20220429AEC001], meanwhile the study was conducted and reported in accordance with the Animal Research: Reporting of In Vivo Experiments (ARRIVE) guidelines.

### Social defeat stress

To create a socially defeated model, reliably aggressive ICR mice (three consecutive attacks within 30 s) were selected as aggressor mice according to a previous report. Briefly, mice in the social defeat and social defeat + PBA groups were physically exposed to a different aggressor for 10 min every day for 10 days. After physical exposure, the defeated mice and aggressors were housed in a shared home cage separated by a clear perforated divider to induce continuous psychological stress (smell and sight of the aggressor) for the remainder of the 24 h (Supplement Fig. 1). Many studies use this model as chronic stress model, and which could cause liver injury by inducing inflammation and apoptosis^18^.

### Preparation of liver tissue and blood sample

After the social avoidance test, blood samples were harvested from the left ventricle under anaesthesia. After being centrifuged for 10 min at 3000 rpm serum, the samples were stored at − 80 °C until analysis. The livers were rapidly removed after the social avoidance test; part of liver tissue randomly taken from the left lobes was kept in 4% paraformaldehyde for histological studies, and the other part was stored at − 80 °C for western blotting.

### Social avoidance test

**Figure 2:**
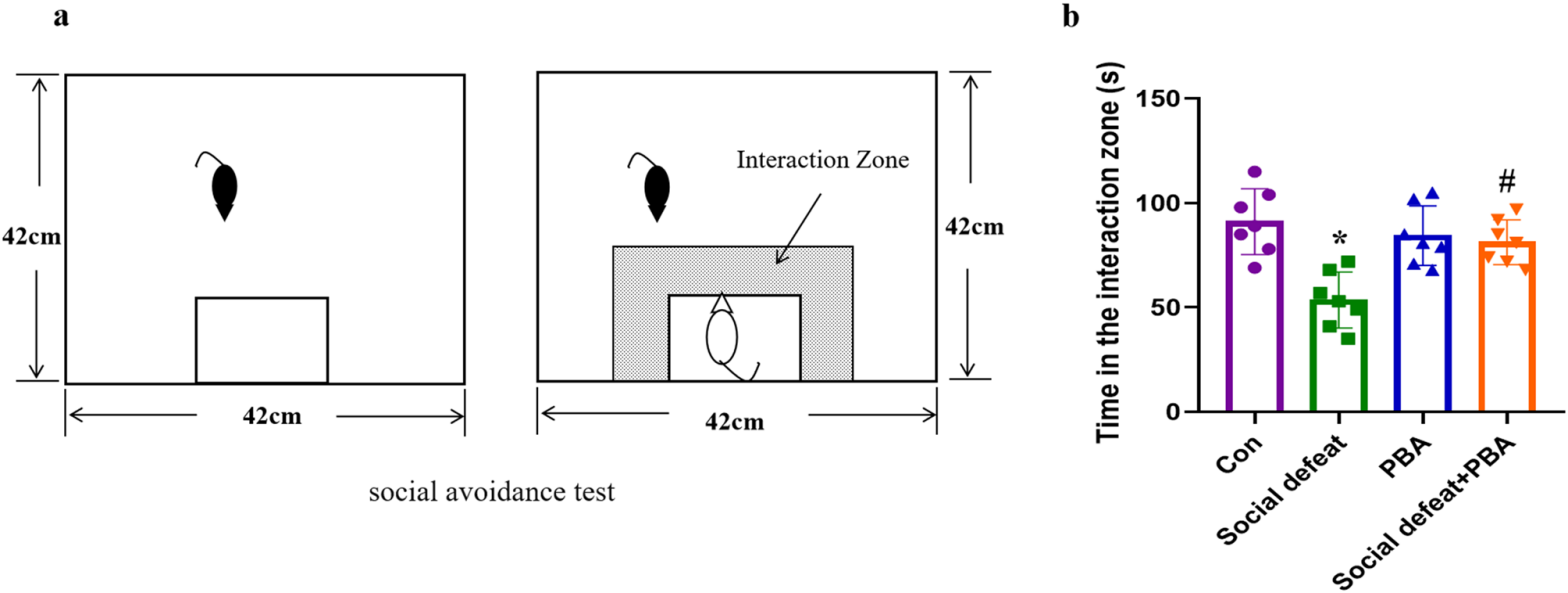
Effects of social defeat on social avoidance test. (**a**) social avoidance text. (**b**) Time in the interaction zone. Data are expressed as mean ± standard deviation. N = 7 per group; *p < 0.01 vs con group; #*p* < 0.01 vs social defeat group. PBA: 4-phenylbutyric acid.

After social defeat stress, the behaviour of the mice was tested using a social avoidance test. The test was conducted in an interactive test box (42 cm × 42 cm × 42 cm) with an empty wire-mesh cage (10 cm × 4.5 cm) located at one end. The mice were placed in the interaction test box and then allowed to adapt for 2.5 min, followed by 2.5 min in the presence of an unfamiliar aggressor (ICR mice) confined in a wire-mesh cage. Each mouse was tested only once. The interaction time (time spent in an interaction zone, which was defined as an eight-cm-wide area surrounding the wire mesh cage) was calculated at the end of the test (Fig. 2a).

### Enzyme‐linked immunosorbent assay (ELISA)

Norepinephrine (NE) levels in the serum were determined using ELISA kits (Bioengineering Institute, Nanjing, Jiancheng, China) as described in the instructions. The absorbance was measured at 450 nm, and the unit was measured in ng/ml. Cholesterol levels in liver tissue were determined using reagent (Fischer Scientifi c, Pittsburgh, USA) with readings at 500 nm, and the unit was measured in mg/dL.

### Hepatotoxicity and oxidative damage

Serum levels of AST and ALT were assayed on colorimetric slides using the Fuji DRI-CHEM system (Fujifilm, Tokyo, Japan) according to the manufacturer’s instructions.

Level of SOD (an antioxidant enzyme) and MDA (a lipid peroxidantion product) in liver homogenates were measured by commercial kits of Nanjing Jiancheng Bioengineering Institute (Nanjing, China), according to the instructions.

### Histological studies

Liver samples were randomly taken from the left lobes, washed with cold phosphate buffer (pH 7.4) and then the 4% paraformaldehyde solution fixed livers were dehydrated in graded ethanol and embedded in paraffin. Next, sections with a thickness of 5–6 um were sliced from the paraffin-embedded blocks. Liver tissue sections were stained with haematoxylin and eosin (H&E), and liver injury was scored on a scale of 0–4 for sinusoidal congestion, vacuolisation of hepatocyte cytoplasm and parenchyma, as described by Suzuki et al.^19^ masson’s trichrome staining (G1003 and G1006, Servicebio, Wuhan, China) was then used for the deposition of collagen fibers in hepatic sections. The fibrosis stage was assessed according to the Ishak score^20^. Periodic acid-Schiff (PAS) staining (GP1008; Servicebio, Wuhan, China) was utilized to detect glycogen deposition and content according to the manufacturer’s instructions.

### Immunofluorescence staining

Liver tissue sliced were incubated in blocking buffer (5% BSA in PBS) for 1 h at room temperature, followed by staining with anti-CD11b, anti-F4/80 (1:500, SERVICEBIO, WuHan, China), anti-Gr-1 (1:100, SANYING, WuHan, China), and anti-p-NF-κB, anti-CHOP (1:200, Abcam, Cambridge, UK). Then sections were incubated with DAPI for nuclei visualization.

### TUNEL staining

Apoptosis was detected using a one-step TUNEL apoptosis assay kit (C1086, Beyotime, Shanghai, China) according to the manufacturer’s instructions. Briefly, 5–6 µm paraffin‐embedded liver sections were dewaxed and rehydrated using a graded series of ethanol (100–70%). The tissue sections were washed with PBS, followed by incubation with the TUNEL reaction mixture (terminal deoxynucleotidyl transferase [TdT] and fluorescein-dUTP) at 37 °C for 1 h in the dark. After washing thrice with PBS, the sections were examined under a light microscope and the apoptotic cell rate was calculated using the following formula: (TUNEL-positive cell number/total area cell number) × 100%.

### Western blotting

Liver tissues were homogenised in 20 mM ice-cold Tris–HCl (pH 7.4), containing 1% protease and phosphatase inhibitors. The homogenates were centrifuged for 15 min at 18,000 g at 4 °C, and the resulting supernatant fractions were employed for western blotting. Equal amounts of protein (30 μg) from liver tissue were loaded onto and transferred to hydrophobic polyvinylidene difluoride membranes. The membranes were blocked with 5% skim milk for 1 h; thereafter, they were incubated overnight at 4 °C with anti-CHOP, anti-p-NF-κB, anti-p-PERK, anti-p-eIF2α, anti-TNFα, anti-cleaved caspase-3 antibody (1:1000; Cell Signaling Technology, Inc., Danvers, MA, USA), anti-PERK, anti-eIF2α, anti-Bax, anti-Bcl-2 antibody (1:500; Santa Cruz Biotechnology, Santa Cruz, CA, USA), anti-FBRS antibody (1:1000; Invitrogen, Carlsbad, CA, USA) or anti-IκBα antibody (1:1000; Abcam, Cambridge, UK) in 5% non-fat milk or 5% bovine serum albumin. The next day, the membranes were washed with phosphate-buffered saline + Tween, and the primary antibody was detected using a horseradish peroxidase-conjugated goat anti-rabbit IgG antibody (1:20,000; Vector, Burlingame, CA, USA) or horseradish peroxidase-conjugated horse anti-mouse IgG antibody (1:5000) in phosphate-buffered saline for 60 min at 25 °C. The blots were developed using an enhanced chemiluminescence reagent (GE Healthcare Inc., Piscataway, NJ, USA). Finally, the blots were visualised via a LAS-3000 Plus lumino-imaging analyser (Fuji Photo Film Company, Kanagawa, Japan) and quantified using Multi Gauge software v3.0 (Fujifilm, Tokyo, Japan).

### Equipment and settings

For Western blot, some membranes were cut before hybridization with primary antibodies and therefore the full membrane image is the size of the cut membrane and sometimes the edges of the membranes will not be clearly visible since Chemidoc images have a light background. Microsoft PowerPoint was used to crop the images to fit the layout of the manuscript.

### Statistics

All data are presented as mean ± standard deviation (SD). The significance of the differences observed among multiple groups was evaluated using a one-way analysis of variance (ANOVA). Post hoc individual comparisons were made using Tukey's honestly significantly different test. A value of *p* < 0.05 was considered statistically significant. Data were analysed using GraphPad Prism version 8.0 software (Graphpad Software Inc).

### Ethical approval

All experiments were conducted in accordance with the guidelines for animal experimentation at Xinxiang Medical University.

## Results

### Verification that the model is related to the experiment

The social avoidance test is an effective way to verify the success of establishing a social defeat stress model. A significant difference among control, social defeat, PBA, and social defeat + PBA groups was observed with regard to spent time in the interaction zone (F(3,28) = 8.902, *p* = 0.0003). Socially defeated mice spent less time in the interaction zone compared to the control group (*p* < 0.01). Administration of PBA significantly increased the time spent in the interaction zone compared with the social defeat group (*p* < 0.01). These findings suggest that the social defeat stress model was successfully established and that PBA altered behavioral changes caused by social defeat stress (Fig. 2b).

### PBA decreases NE concentration, metabolic parameters of liver, and attenuates social defeat stress-induced liver injury

**Figure 3:**
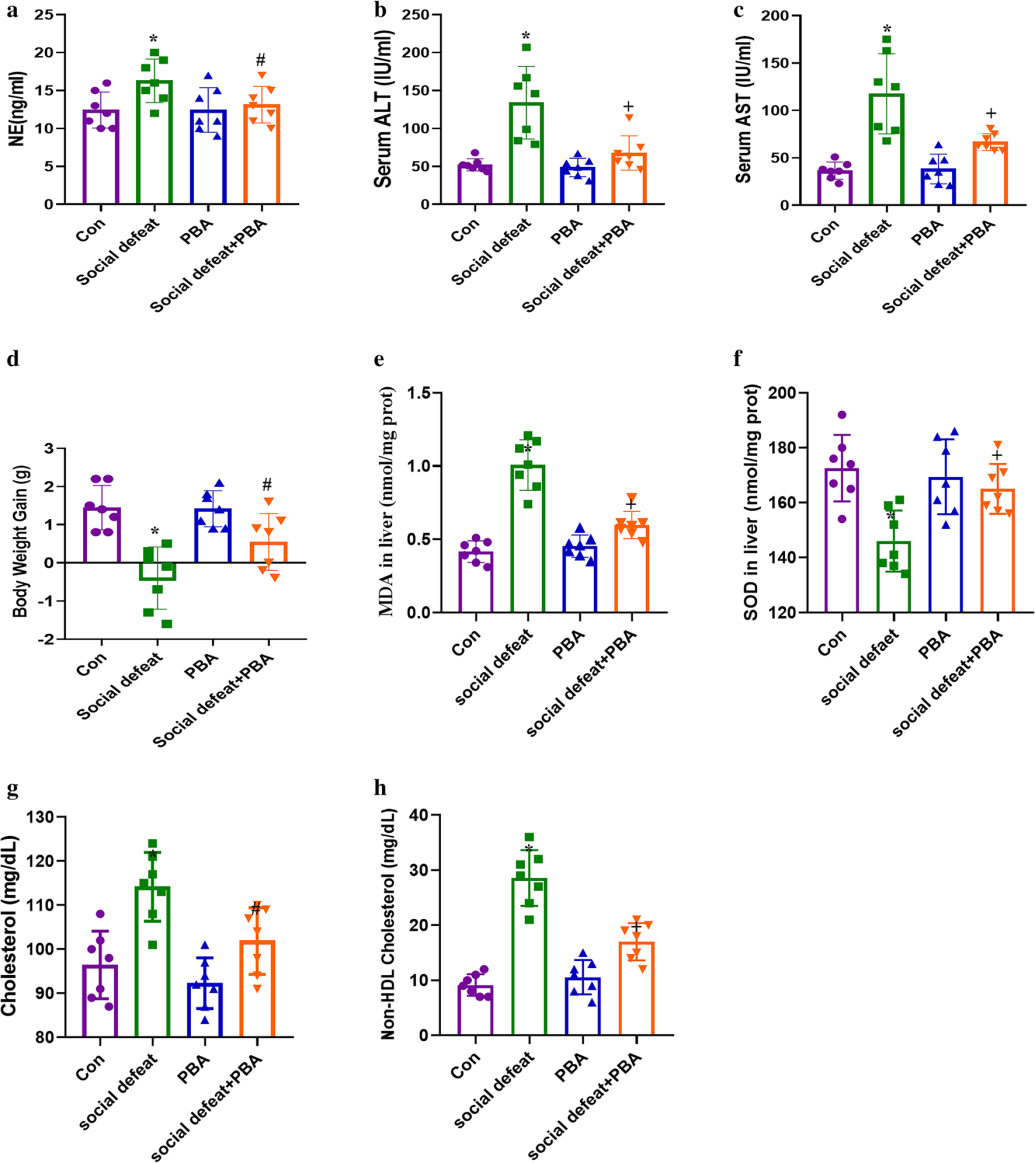
Effects of PBA on NE concentration, ALT and AST activity, MDA and SOD level. (**a**) NE concentration in serum. Activity of ALT (**b**) and AST (**c**) in serum. (**d**) Body weight gain of each group is presented. Level of MDA (**e**) and SOD (**f**) in liver sample. Level of total cholesterol (**l**) and non-HDL cholesterol (**i**) in liver sample. Data are expressed as mean ± standard deviation. N = 7 per group; *p < 0.01 vs con group; #*p* < 0.05 vs social defeat group; + *p* < 0.01 vs social defeat group. PBA: 4-phenylbutyric acid.

**Figure 4:**
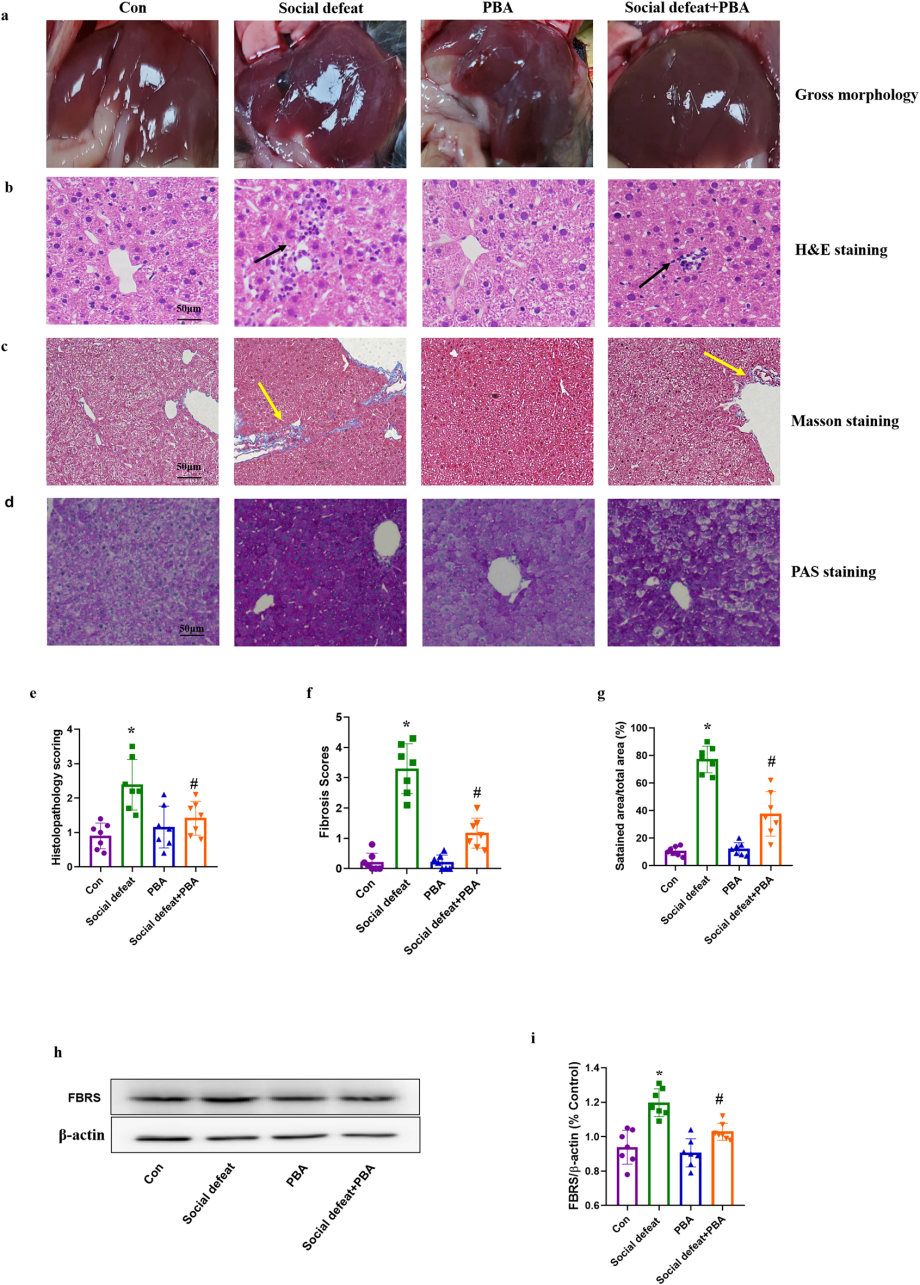
Effects of PBA on social defeat stress-induced liver injury. (**a**) Gross morphology of liver. (**b**) H&E staining (×200), (**c**) Masson staining (×100) and (**d**) PAS staining (×100). Representative images of each group are presented. (**e**) Histopathological mean liver injury scores in (**b**). (**f**) Quantification for fibrotic areas by Masson staining in (**c**). (**g**) Quantification of PAS-stained area in (**d**). (**h**) The protein expression of FBRS and the bar graph summarizes ratio of protein expression of FBRS/β-actin (**i**). Black arrows indicate inflammatory cell infiltration. Yellow arrows indicate fibrotic in liver tissue. Data are expressed as mean ± standard deviation. N = 7 per group; *p < 0.01 vs con group; #*p* < 0.05 vs social defeat group. PBA: 4-phenylbutyric acid.

The concentration of NE, ALT, AST, MDA, SOD, cholesterol, non-HDL cholesterol and body weight gain were showed in Fig. 4. Concentration of NE (F(3,24) = 3.428, *p* = 0.0331), ALT (F(3,24) = 14.71, *p* < 0.01), AST (F(3,24) = 18.28, *p* < 0.01), MDA (F(3, 24) = 41.58, *p* < 0.01), SOD (F(3, 24) = 41.58, *p* = 0.01), cholesterol (F (3, 24) = 20.59, *p* < 0.01), non-HDL cholesterol (F(3, 24) = 49.91, *p* < 0.01) and body weight gain (F(3,24) = 11.96, *p* < 0.01) varied significantly among four groups. NE concentration was significantly higher in the social defeat stress group as compared to the control group (Fig. 3a, *p* < 0.05), which was attenuated by treatment with PBA. Serum ALT (Fig. 3b, *p* < 0.01) and AST (Fig. 3c, *p* < 0.01) levels in the social defeat stress-induced mice were significantly higher in comparison to control mice. Furthermore, administration of PBA markedly alleviated the increase in serum enzyme activity, indicating that PBA has a benefit in social defeat stress-induced liver injury. However, the administration of PBA alone did not affect the activities of ALT and AST. Compared with the control group, the social defeat group showed lower body weight gain (Fig. 3d, *p* < 0.01), which was recorded after treatment with PBA. Meanwhile, Social defeat stress increased lipid peroxidation products and decreased activities of antioxidant enzymes. The level of MDA (Fig. 3e, *p* < 0.01) was significantly increased and SOD (Fig. 3f, *p* < 0.01) was dramatically decreased in the liver of mice in the social defeat stress group compared to the control group, which was alleviated by administration of PBA. Furthermore, we noticed that social defeat stress affect lipid profiles. The level of total cholesterol (Fig. 3g, *p* < 0.01) and non-HDL cholesterol (Fig. 3h, *p* < 0.01) in the social defeat stress-induced mice were significantly higher in comparison to control mice. Liver cysts were found in the social defeat group by gross morphological examination (Fig. 4a). Histopathological changes are a direct indication of liver injury. H&E staining of liver tissue in the control group showed structured liver lobules clearly. However, in the social defeat stress group, changes were observed in live tissue, including vascular degeneration and inflammatory cell infiltration (Fig. 4b), and the histopathological mean liver injury scores were much higher in the social defeat group than in the control group (Fig. 4e, *p* < 0.01). Administration of PBA significantly attenuated the social defeat stress-induced liver damage (Fig. 4b, e, *p* < 0.05). Meanwhile, social defeat stress led to liver fibrosis and abnormal glycogen deposition, which were attenuated by the administration of PBA (Fig. 4c and d). The fibrosis scores (Fig. 4f, F(3,24) = 55.48, *p* < 0.01) and PAS-stained areas (Fig. 4g, F(3,24) = 69.91, *p* < 0.01) were all significantly higher in the social defeat group than in the control group and were reduced by administration of PBA. Furthermore, we detected the level of fibrosin (FBRS) in four groups (Fig. 4i, F(3, 24) = 18.75, *p* < 0.01), which was dramatically increased in social defeat group, and was recorded by administration of PBA (Fig. 4h and i).

### PBA prevents social defeat stress-induced ER stress in liver

**Figure 5:**
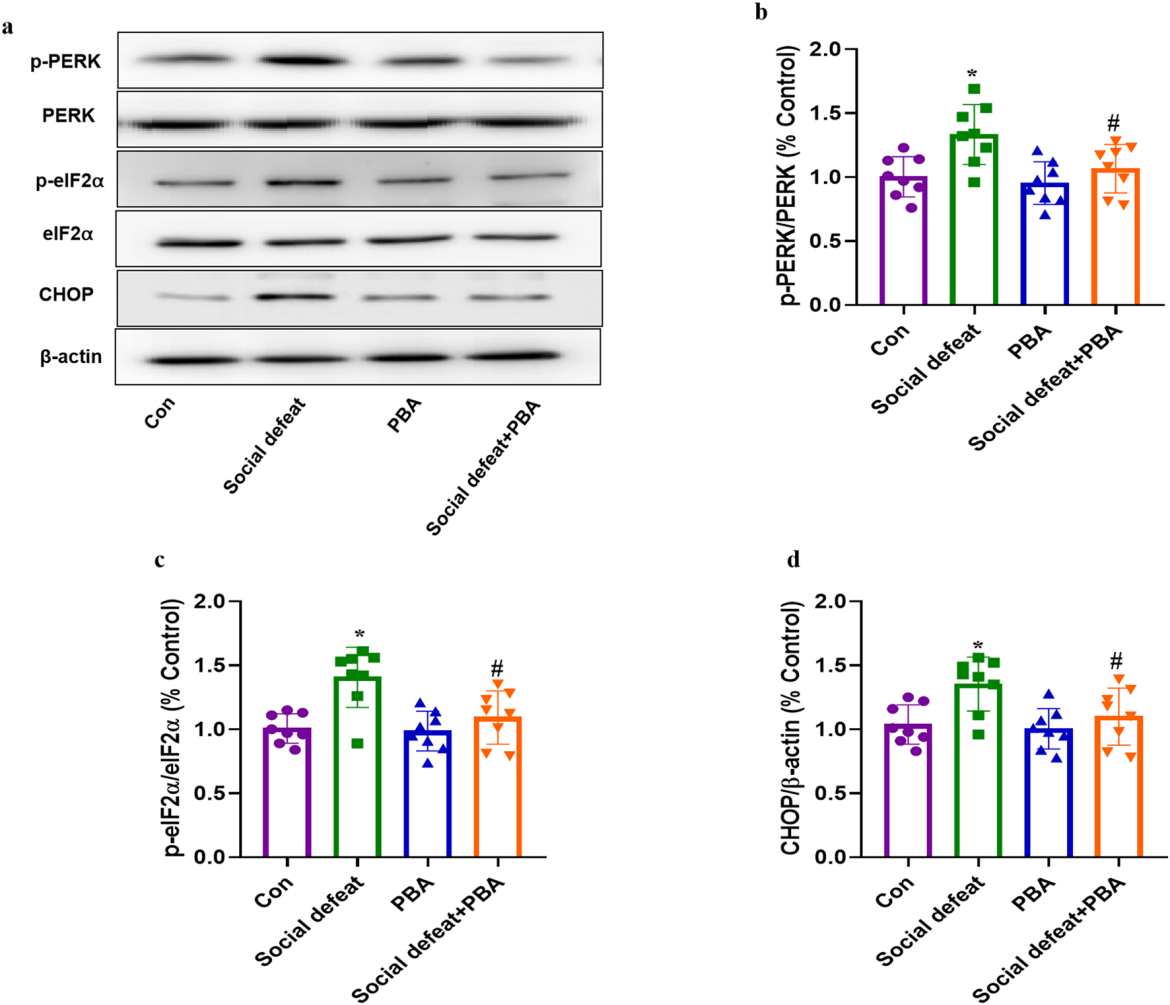
Effects of PBA on ER stress marker levels in the liver induced by social defeat stress. (**a**) The protein expression of p-PERK, p-IF2⍺ and CHOP and the bar graph summarizes the ratio of protein expression of p-PERK/PERK (**b**), p-eIF2⍺/eIF2⍺ (**c**) and CHOP/β-actin among groups. Data are expressed as mean ± standard deviation. N = 8 per group; *p < 0.01 vs con group; #*p* < 0.05 vs social defeat group. PBA: 4-phenylbutyric acid.

To determine the role of ER stress in the effects of social defeat stress on liver injury, we measured the levels of p-PERK, p-eIF2α and CHOP, which are major cellular markers of ER stress, in liver tissues from four groups of mice (F[3, 28] = 5.628, *p* < 0.01). Prolonged ER stress contributes to liver apoptosis by increasing p-PERK, p-eIF2α and CHOP expression levels. Western blotting revealed significantly increased p-PERK (Fig. 5a and b, *p* < 0.01), p-eIF2α (Fig. 5a and c, *p* < 0.01) and CHOP (Fig. 5a and d, *p* < 0.01) expression in liver tissues from the social defeat group compared to the control group. PBA treatment reversed these effects (Fig. 5a, b, c, and d). However, the administration of PBA alone did not influence the expression of p-PERK, p-eIF2α and CHOP. Hence, social defeat stress induced ER stress in the liver tissues of mice, which was attenuated by PBA.

### PBA reduces liver inflammation induced by social defeat stress

**Figure 6:**
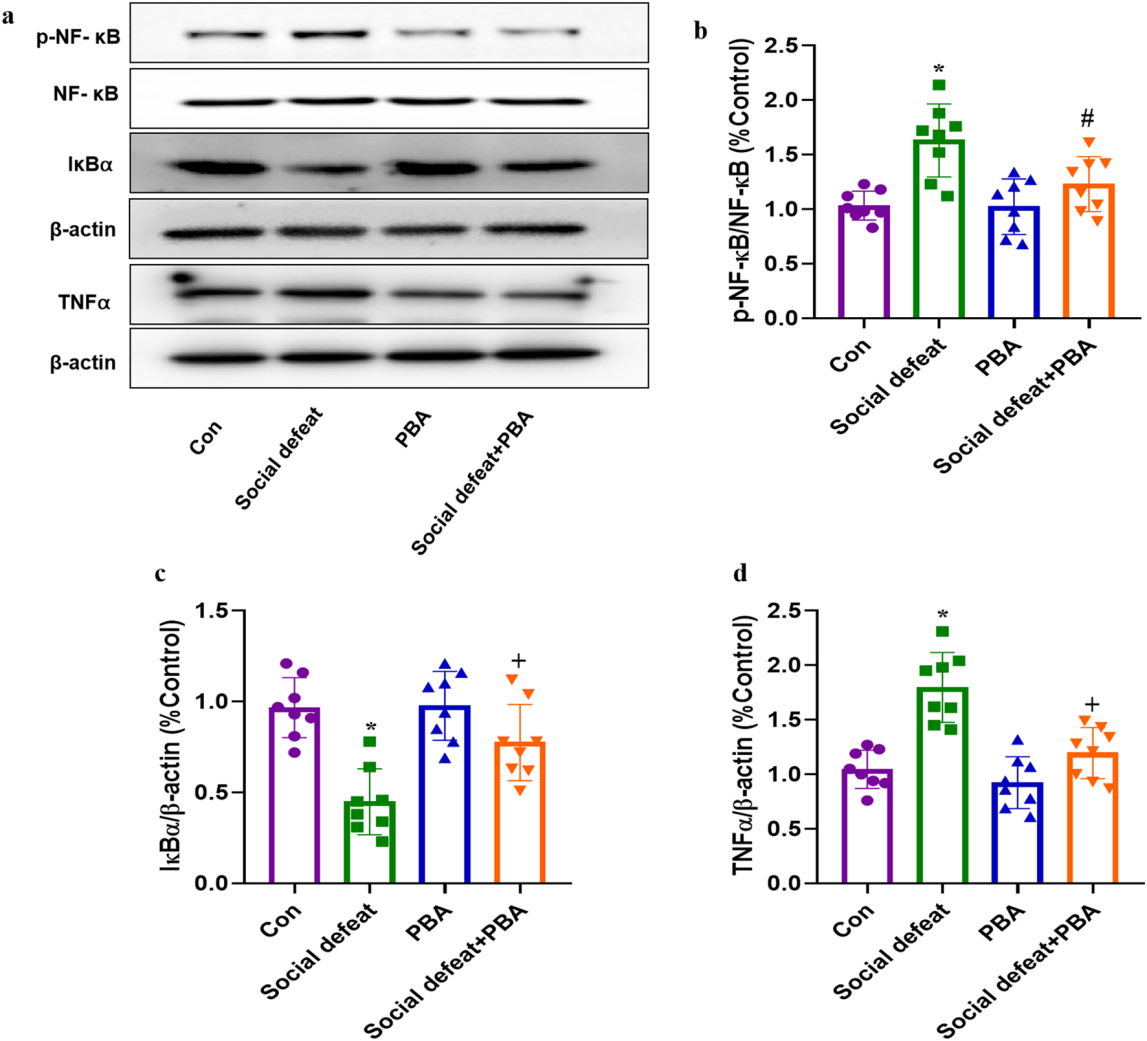
Effects of PBA on inflammatory factors in the liver induced by social defeat stress. (**a**) The protein expression of p-NF-κB, IκBα and TNFα and the bar graph summarizes the ratio of protein expression of p-NF-κB/NF-κB (**b**), IκBα/ β-actin(**c**) and TNFα/β-actin (**d**) among groups. Data are expressed as mean ± standard deviation. N = 8 per group; *p < 0.01 vs con group; #*p* < 0.05 vs social defeat group; +*p* < 0.01 vs social defeat group. PBA: 4-phenylbutyric acid.

NF-κB is a DNA-binding protein that modulates ER stress and inflammatory responses, both of which are vital in liver injury. NF-κB phosphorylation results in the overexpression of proinflammatory cytokines, including TNFα. Western blot analysis revealed that the expression of p-NF-κB (Fig. 6a and b, F[3, 28] = 10.03, *p* < 0.01) and TNFα (Fig. 6a and d, F[3, 28] = 19.62, *p* < 0.01) differed significantly among the four groups. The expression of p-NF-κB (Fig. 6b, *p* < 0.01) and TNFα (Fig. 6d, *p* < 0.01) increased in mice exposed to social defeat stress. However, treatment with PBA attenuated the social defeat stress-induced increase in p-NF-κB expression compared to the social defeat only group (Fig. 6a and b, *p* < 0.05), suggesting that ER stress has an important role in the observed effects. In contrast, the expression of IκBα (Fig. 6c, *p* < 0.01), which inhibits the transcription of NF-κB-dependent pro-inflammatory and apoptotic genes, was significantly decreased in the social defeat group compared to the control group. These effects were prevented by PBA treatment; however, treatment with PBA alone did not affect the expression of p-NF-κB, TNF-α and IκBα.

### PBA attenuated social defeat stress induced inflammatory cell infiltration and immune response in the liver of mice

**Figure 7:**
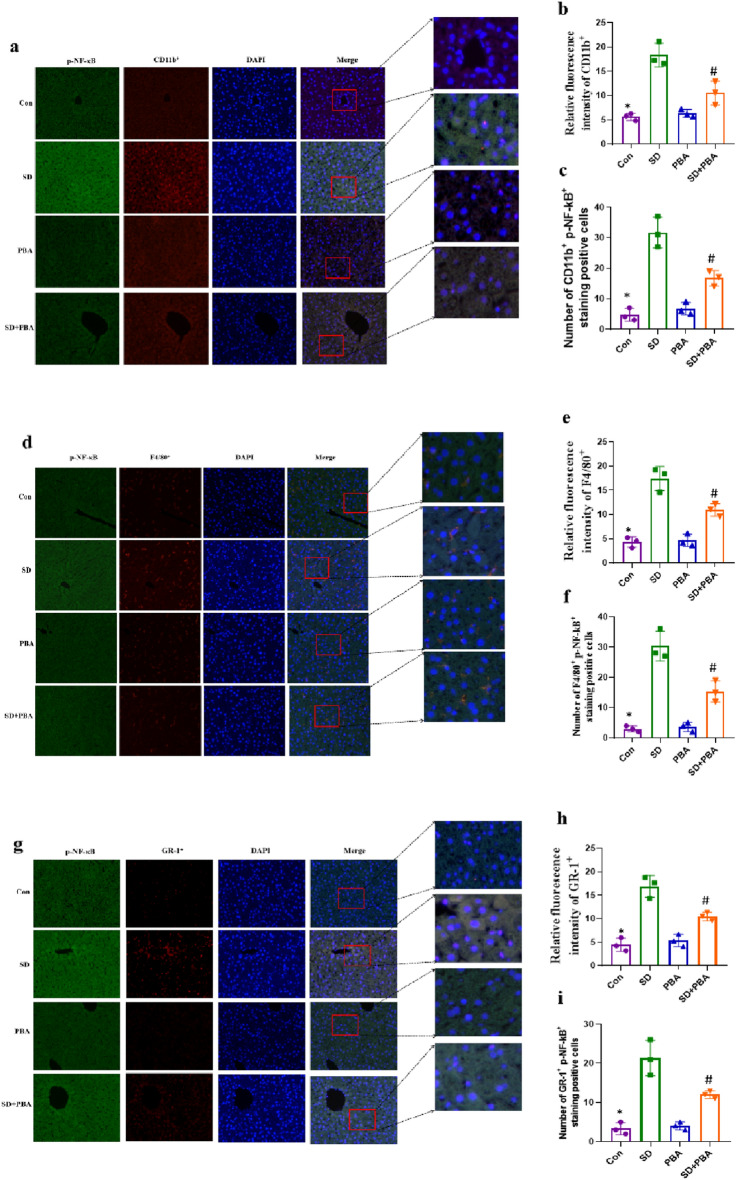
Effects of PBA on inflammatory cell infiltration in the liver induced by social defeat stress. (**a**) Immunofluorescent staining of CD11b^+^ macrophages, F4/80^+^ macrophages, Gr-1þ^+^ neutrophils and CHOP in the liver were shown (×100). Relative fluorescence intensity of CD11b^+^ macrophages (**b**), F4/80^+^ macrophages (**e**), and Gr-1^+^ neutrophils (**i**) were determined. Number of p-NF-κB and CD11b^+^ positive cells (**c**). Number of p-NF-κB and F4/80^+^ positive cells (**f**). Number of p-NF-κB and Gr-1^+^ positive cells (**j**). *p < 0.01 vs con group; #*p* < 0.01 vs social defeat stress group. PBA: 4-phenylbutyric acid, SD: social defeat.

To evaluated the expression of p-NF-κB and CHOP in Kupffer cells, immunofluorescence assay was used in this study. As shown in Fig. 7 and supplement Fig. 2 social defeat stress increase of CD11b^+^ macrophages, F4/80^+^ macrophages, and Gr-1^+^ neutrophils in the liver was dramatically reduced (*p* < 0.05) by PBA administration. Furthermore, we examined the expression of p-NF-κB (Fig. 7a, d, g, *p* < 0.01) and CHOP (Supplement Fig. 2a, d, g, *p* < 0.01) by immunofluorescence staining. Indeed, social defeat stress increased macrophages and neutrophils p-NF-κB (Fig. 7b, c, e, f, h, i) and CHOP (Supplement Fig. 2b, c, e, f, h, i) expression compared with control, and which was also reversed by treatment with PBA.

### PBA ameliorates social defeat stress-induced hepatocyte apoptosis

**Figure 8:**
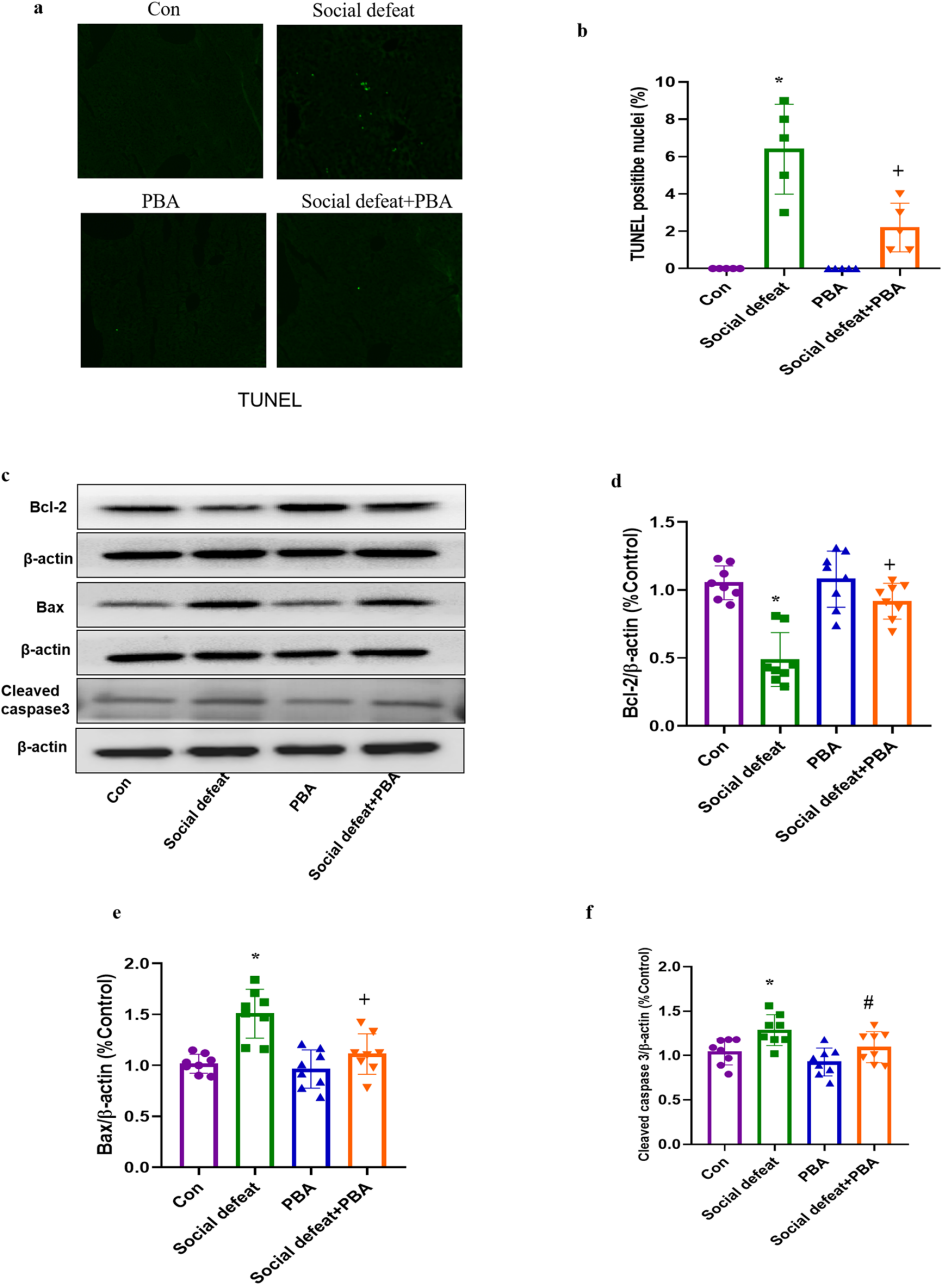
Effects of PBA on apoptosis in the liver induced by social defeat stress. (**a**, **b**) Representative images and quantification of TUNEL-stained liver sections. (**c**) The protein expression of Bcl-2, Bax and TNFα and the bar graph summarizes the ratio of protein expression of Bcl-2/β-actin (**d**), Bax/ β-actin(**e**) and cleaved caspase 3/β-actin (**f**) among groups. Data are expressed as mean ± standard deviation. N = 5 or 8 per group; *p < 0.01 vs con group; #*p* < 0.05 vs social defeat group; +*p* < 0.01 vs social defeat group. PBA: 4-phenylbutyric acid.

Apoptosis is a prominent feature of liver diseases that we examined in the liver tissues of socially defeated or PBA-treated mice. The TUNEL test showed that social defeat stress induced remarkable TUNEL-positive cells (Fig. 8a and b, *p* < 0.01), which were restored significantly by PBA treatment (Fig. 8a and b, *p* < 0.01). To further confirm the protective role of PBA in social defeat stress-induced hepatocyte apoptosis, we measured the levels of the apoptosis-related proteins Bcl-2 (F[3, 28] = 20.78, *p* < 0.01), Bax (F[3, 28] = 13.74, *p* < 0.01) and cleaved caspase-3 (F[3, 28] = 6.756, *p* < 0.01) in the four groups (Fig. 8c, d, e, and f). Our results showed that social defeat stress reduced Bcl-2 (Fig. 8c and d, *p* < 0.01), increased Bax (Fig. 8c, and e, *p* < 0.01) and cleaved caspase-3 (Fig. 8c and f, *p* < 0.01) levels compared with the control group, which was alleviated by treatment with PBA. However, PBA treatment alone did not impact the expression of Bcl-2, Bax or cleaved caspase-3. These findings suggest that PBA inhibits apoptosis in the liver.

### PBA improves TG-induced ER stress-related inflammation and apoptosis in liver injury

**Figure 9:**
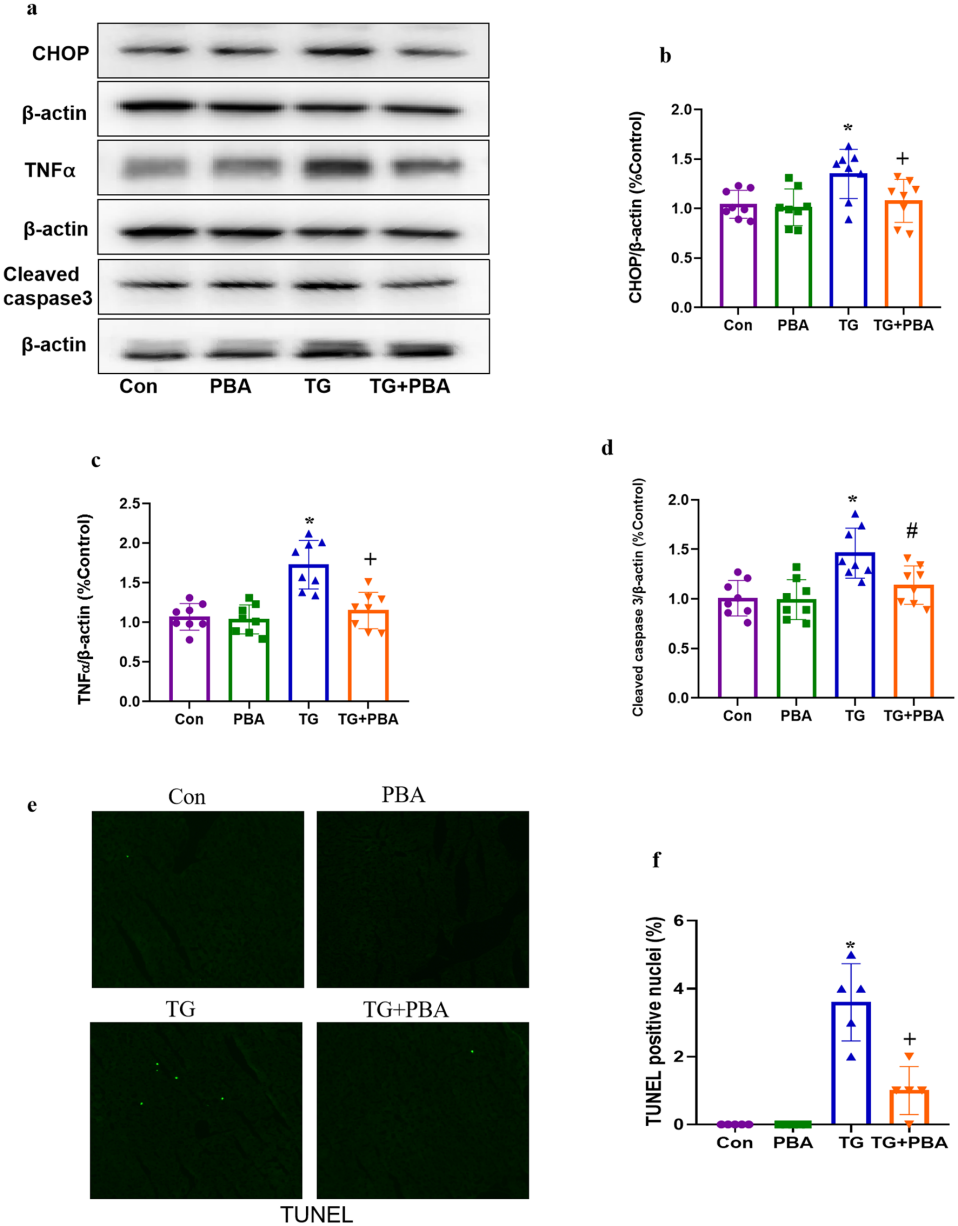
Effects of PBA on TG induced-ER stress-related inflammation and apoptosis. (**a**) The protein expression of CHOP, TNFα and cleaved caspase 3 and the bar graph summarizes the ratio of protein expression of CHOP/β-actin (**b**), TNFα/β-actin (**c**) and cleaved caspase 3/β-actin (**d**) among groups. (**e**, **f**) Representative images and quantification of TUNEL-stained liver sections. Data are expressed as mean ± standard deviation. N = 5 or 8 per group; *p < 0.01 vs con group and PBA group; #*p* < 0.05 vs TG group; +*p* < 0.01 vs TG group. PBA: 4-phenylbutyric acid; TG: Thapsigargin.

To confirm the role of ER stress in the expression of inflammatory and apoptotic factors, we treated mice with TG to induce ER stress. Western blot analysis revealed that the expression of CHOP (F(3,28) = 4.667, *p* = 0.0091), TNFα (F(3,28) = 16.09, *p* < 0.01) and cleaved caspase-3 (F(3,28) = 8.761, *p* = 0.0003) differed significantly among the four groups. The Administration of TG dramatically increased CHOP protein expression (Fig. 9a and b, *p* < 0.01) and then increased the expression of TNFα (Fig. 9a and c, *p* < 0.01) and cleaved caspase-3 (Fig. 9a and d, p < 0.01), which was attenuated by pre-treatment with PBA. However, treatment with PBA alone did not affect CHOP, TNFα or cleaved caspase-3 expression. These outcomes denote that TG-induced ER stress increased TNFα and cleaved caspase-3 expression, which was alleviated by PBA. Meanwhile, the administration of TG increased the number of TUNEL-positive cells (Fig. 9e and f, *p* < 0.01), which was alleviated by PBA (Fig. 9e and f, *p* < 0.01).

### PBA decreases ALT and AST activity and attenuates TG-induced liver injury

To verify the findings of PBA attenuates liver injury by inhibit ER stress, we examined the the level of ALT, AST, H&E, Masson and PAS staining among control, PBA, TG, and PBA + TG four groups. Concentration of ALT (F(3,24) = 15.37, *p* < 0.01), AST (F(3,24) = 11.44, *p* < 0.01) histopathological mean liver injury scores (F(3,24) = 12.33, *p* < 0.01), fibrosis scores (F(3,24) = 42.22, *p* < 0.01) and PAS-stained areas (F(3,24) = 73.2, *p* < 0.01) varied significantly among four groups. Administration of TG significantly increased serum ALT (Supplement Fig. 3a, *p* < 0.01) and AST (Supplement Fig. 3b, *p* < 0.01) levels, which were attenuated by PBA (Supplement Fig. 3a and b, p< 0.01, or *p* < 0.01). Liver cysts were found in the TG group on gross morphological examination (Supplement Fig. 3c). H&E staining showed TG-induced balloon degeneration, inflammatory cell infiltration and haemorrhage (Supplement Fig. 3d). Meanwhile, the histopathological mean liver injury scores were higher in the TG group than in the control group (Supplement Fig. 3g, *p* < 0.01). Masson and PAS staining indicated that TG increased liver fibrosis and glycogen content compared to the control group (Supplement Fig. 3e and f). The fibrosis scores (Supplement Fig. 3h, *p* < 0.01) and PAS-stained areas (Supplement Fig. 3i, *p* < 0.01) were significantly higher in the TG group in comparison to the control group. Treatment with PBA and TG dramatically decreased TG-induced liver injury; however, administration of PBA alone did not affect ALT and AST activity or liver structure (Supplement Fig. 3).

## Discussion

Previous studies have shown that different kinds of stress, either emotional or social, have damaging effects on cellular tissue integrity^1,3^. Once subjected to a stressor, specific pathways within the brain lead to the release of key peripheral mediators, including catecholamines. NE, a kind of catecholamine, is associated with psychological stress and is released during stress reactions^4^. Some studies have indicated that psychological stress may cause liver damage by regulating hormone changes, liver metabolism and immune response in the body, which is not a direct cause, and changes in hormone levels have physiological significance^10,21,22^. In this study, social defeat stress increased NE levels and reduced the time spent in the interaction zone. These results indicate that the social defeat stress model is successful, and hormonal changes may be involved in the stress that causes liver damage. Administration of PBA reduced NE expression and increased the time spent in the interaction zone.

ALT and AST are sensitive indicators of liver cell injury and help recognize hepatocellular diseases. These enzymes change in response to catecholamine release^23^. In this study, social defeat stress increased serum ALT and AST levels and caused hepatic architecture damage, including inflammatory cell infiltration and vascular degeneration, which were detected by H&E staining. Meanwhile, social defeat stress increased liver fibrosis and abnormal glycogen deposition, as examined by Masson and PAS staining. PBA treatment effectively decreased serum ALT and AST levels and reversed liver injury in a social defeat stress-induced mouse model. Moreover, social defeat stress affect lipid profiles and oxidative stress, the level of MDA, cholesterol, and non-HDL cholesterol was increased and the level of SOD was decreased, which was reversed by treatment of PBA. These results indicating social defeat stress disrupts regulation of lipid synthesis and cause oxidative damage in the liver. Further research is needed to investigate the mechanism underlying the protective effects of PBA against liver injury caused by social failure stress.

The ER has a critical role in protein synthesis and folding. Several studies have indicated that ER stress-related proteins are involved in cellular injury in various tissues, including hepatocytes and cardiomyocytes^24,25^. Our previous studies demonstrated that CHOP and GRP78 expression were significantly and positively correlated with social defeat stress-induced cognitive dysfunction and myocardial injury. Recent studies have demonstrated that noise pollution or forced swimming affects emotional status and leads to cell injury in liver tissues^9^. However, the precise role of ER stress in the liver after social defeat stress is yet to be demonstrated. PERK, eIF2α and CHOP are the key factors involved in ER stress^3^. It has been reported that activated PERK, eIF2α and CHOP mediate hepatocyte necroptosis in acute liver injury. In this study, the social defeat group exhibited elevated expression of p-PERK, p-eIF2α and CHOP, and caused liver injury compared to the control group. Administration of PBA prevented adverse changes in p-PERK, p-eIF2α and CHOP expression and reduced subsequent liver damage induced by social defeat stress. These results indicated that ER stress is essential in social defeat stress-induced liver injury. Moreover, inhibition of ER stress is beneficial in the livers of socially defeated mice.

The IKK/NF-κB signaling pathway is a well-known mediator of inflammatory and stress responses^26^. Exposure to stress induces IκBα phosphorylation, leading to IκBα degradation and NF-κB activation,when activated, NF-κB translocates to the nucleus, binds the DNA and regulates the expression of over 200 different genes. The product of these genes regulate the immune system and inflammation^27^. The NF-κB signaling pathway is also related to ER stress and liver injury. Additionally, TNF-α is an activator of the NF-κB pathway and is regulated by NF-κB^28^. A study showed that the protective effect of PBA in EtOH-induced liver injury was reported to occur partly by inhibition of TNFα activity^29^. This study demonstrated that social defeat stress significantly increased NF-κB phosphorylation and IκBα degradation, which resulted in increased TNF-α protein expression in social defeat stress-induced liver injury. PBA treatment reversed these effects. Thus, PBA reduced inflammation partly via inhibition of the NF-κB pathway in social defeat stress-induced liver injury.

Liver macrophages (Kupffer cells) are generally in the resting state. Upon stimulation by pathogens or cytokines, they can be activated and have an enhanced function. Meanwhile, Kupffer cells, the key components of the hepatic innate immune system, represent the first line of defence in detecting the invading pathogens in the liver. Activated macrophages leading the production of NF-κB, TNF-α and IL-6 which promote the up-regulation of adhesion molecules on the luminal site of endothelial cells to aid with migration of neutrophils^30–32^. In this study, we detected social defeat stress increased immune cell infiltration, meanwhile, the expression of p-NF-κB and CHOP was increased in macrophages. So, Kupffer cells may play an important role in social defeat stress induced liver injury, however, further research is needed to investigate other liver cell types response to the stress.

Apoptosis is a genetically regulated form of cell death with important physiological significance^32,33^ Apoptosis may be linked to prolonged ER stress in liver injury. Enhanced ER stress induces apoptosis via the CHOP pathway^6^. The caspase family of cysteine proteases is a key mediator of programmed cell death and apoptosis. Cleaved caspase‐3 is important for the caspase cascade; once activated, it activates the rest of the apoptotic pathway. Furthermore, the anti-apoptotic protein Bcl-2 and the pro-apoptotic protein Bax, which have an essential role in the mitochondrial pathway of apoptosis, were investigated^34^. This study indicated that PBA administration reversed the up-regulation of CHOP, cleaved caspase-3 and Bax expression while recovering the down-regulation of Bcl-2 expression in social defeat stress-induced liver injury in mice. To further confirm these results, a TUNEL assay was used to detect apoptosis in liver tissue. As expected, social defeat stress induced a significant increase in liver TUNEL-positive cells, which was reduced by treatment with PBA. Therefore, the results indicate that PBA can attenuate liver cell apoptosis caused by social defeat stress.

ER stress has been implicated in acute and chronic liver injury and can cause inflammation and induce apoptosis. We further investigated the relationship between ER stress, inflammation, and apoptosis in liver injury caused by social defeat stress. We treated mice with the ER stress agonist, TG, to induce ER stress. Administration of TG dramatically increased CHOP expression and further increased TNFα and cleaved caspase-3 expression in the liver. However, treatment with PBA alone did not affect the expression of CHOP, TNFα and cleaved caspase-3. Pre-treatment with PBA attenuated the TG-induced increase in the expression of CHOP, TNFα and cleaved caspase-3 in the liver. These findings confirm that TG-induced ER stress further activated inflammation and apoptosis, both of which were attenuated by PBA.

## Conclusion

The results of this study revealed that social defeat stress induces ER stress and promotes inflammation and apoptosis in the liver. Administration of PBA improved hepatic injury by inhibiting the classical NF-κB pathway and regulating the expression of apoptosis-related proteins Bcl-2, Bax and cleaved caspase-3.

## Limitation

Although ER stress, inflammation, apoptosis, and liver injury in mice had been investigated, however, explore level of NF-κB and ER stress in different kinds of liver cells may justice to the hepatic tissue homeostasis both at the metabolic and immune levels better.

### Supplementary Information

Supplementary Information.

## Data Availability

The datasets used and/or analyzed during the current study are available from the corresponding author on reasonable request.

## References

[1] Kim SH, Oh DS, Oh JY, Son TG, Yuk DY, Jung YS (2016). Silymarin prevents restraint stress-induced acute liver injury by ameliorating oxidative stress and reducing inflammatory response. Molecules.

[2] Bauer ME, Jeckel CM, Luz C (2009). The role of stress factors during aging of the immune system. Ann. N. Y. Acad. Sci..

[3] Huang GB, Zhao T, Muna SS, Bagalkot TR, Jin HM, Chae HJ (2013). Effects of chronic social defeat stress on behaviour, endoplasmic reticulum proteins and choline acetyltransferase in adolescent mice. Int. J. Neuropsychopharmacol..

[4] Swain MGI (2000). Stress and hepatic inflammation. Am. J. Physiol. Gastrointest. Liver Physiol..

[5] Lebeaupin C, Vallee D, Hazari Y, Hetz C, Chevet E, Bailly-Maitre B (2018). Endoplasmic reticulum stress signalling and the pathogenesis of non-alcoholic fatty liver disease. J. Hepatol..

[6] Liu XY, Green RM (2019). Endoplasmic reticulum stress and liver diseases. Liver Res..

[7] Li C, Sheng M, Lin Y, Xu DW, Tian YJ, Zhan YQ (2021). Functional crosstalk between myeloid Foxo1-beta-catenin axis and Hedgehog/Gli1 signaling in oxidative stress response. Cell Death Differ..

[8] Zhao T, Huang GB, Muna SS, Bagalkot TR, Jin HM, Chae HJ (2013). Effects of chronic social defeat stress on behavior and choline acetyltransferase, 78-kDa glucose-regulated protein, and CCAAT/enhancer-binding protein (C/EBP) homologous protein in adult mice. Psychopharmacology.

[9] Meng XX, Gu ZH, Xie XP, Su YT, Zhang X, Ma HZ (2019). Acid sphingomyelinase mediates the noise-induced liver disorder in mice. Clin. Exp. Pharmacol. Physiol..

[10] Sha JC, Feng XJ, Chen YP, Zhang HY, Li B, Hu XY (2019). Dexmedetomidine improves acute stress-induced liver injury in rats by regulating MKP-1, inhibiting NF-κB pathway and cell apoptosis. J. Cell Physiol..

[11] Tian RD, Chen YQ, He YH, Tang YJ, Chen GM, Yang FW (2020). Phosphorylation of eIF2α mitigates endoplasmic reticulum stress and hepatocyte necroptosis in acute liver injury. Ann. Hepatol..

[12] Anuncibay SB, Perez RD, Santos GM, Font BE, Ugidos IF, Gonzalez RP (2018). Salubrinal and robenacoxib treatment after global cerebral ischemia. Exploring the interactions between ER stress and inflammation. Biochem. Pharmacol..

[13] Gao B, Zhang XY, Han R, Zhang TT, Chen C, Qin ZH (2013). The endoplasmic reticulum stress inhibitor salubrinal inhibits the activation of autophagy and neuroprotection induced by brain ischemic preconditioning. Acta Pharmacol. Sin..

[14] Dong Y, Liu Y, Kou X, Jing Y, Sun K, Sheng D (2016). The protective or damaging effect of tumor necrosis factor-alpha in acute liver injury is concentration dependent. Cell Biosci..

[15] Feng B, Huang X, Jiang D, Hua L, Zhuo YW (2017). Endoplasmic reticulum stress inducer tunicamycin alters hepatic energy homeostasis in mice. Int. J. Mol. Sci..

[16] Ma J, Luo T, Zeng Z, Fu HY, Asano Y, Liao YL (2016). Histone deacetylase inhibitor phenylbutyrate exaggerates heart failure in pressure overloaded mice independently of HDAC inhibition. Sci. Rep..

[17] Spitler KM, Webb RC (2014). Endoplasmic reticulum stress contributes to aortic stiffening via proapoptotic and fibrotic signaling mechanisms. Hypertension.

[18] Aarti G, Peter DA, Duncan ED, Muhie S, Chakraborty N, Luke BT (2015). Acute and chronic plasma metabolomic and liver transcriptomic stress effects in a mouse model with features of post-traumatic stress disorder. PLOS ONE.

[19] Suzuki S, Nakamura S, Koizumi T, Sakaguchi S, Baba S, Muro H (1991). The beneficial effect of a prostaglandin I2 analog on ischemic rat liver. Transplantation.

[20] Polasek M, Fuchs BC, Uppal R, Schuhle DT, Alford JK, Loving GS (2012). Molecular MR imaging of liver fibrosis: A feasibility study using rat and mouse models. J. Hepatol..

[21] Barbieri A, Bimonte S, Palma G, Luciano A, Rea D, Giudice A (2015). The stress hormone norepinephrine increases migration of prostate cancer cells in vitro and in vivo. Int. J. Oncol..

[22] Kim HG, Jin SL, Lee JS, Han JM, Chang GS (2012). Hepatoprotective and antioxidant effects of Myelophil on restraint stress-induced liver injury in BALB/c mice. J. Ethnopharmacol..

[23] McGill MR (2016). The past and present of serum aminotransferases and the future of liver injury biomarkers. EXCLI J..

[24] Doroudgar S, Volkers M, Thuerauf DJ, Khan M, Mohsin S, Respress JL (2015). Hrd1 and ER-associated protein degradation, ERAD, are critical elements of the adaptive ER stress response in cardiac myocytes. Circ. Res..

[25] Pagliassotti MJ (2012). Endoplasmic reticulum stress in nonalcoholic fatty liver disease. Annu. Rev. Nutr..

[26] Judith SS, Melanie M, Aladdin A, Pavel S, Alina E, Ephraim AM (2023). NF-κB is a critical mediator of post-mitotic senescence in oligodendrocytes and subsequent white matter loss. Mol. Neurodegener..

[27] Younes A, Garg A, Aggarwal BB (2003). Nuclear transcription factor-kappaB in Hodgkin’s disease. Leuk. Lymphoma.

[28] He M, Wang C, Sun JH, Liu Y, Wang H, Zhao JS (2017). Roscovitine attenuates intimal hyperplasia via inhibiting NF-kappaB and STAT3 activation induced by TNF-alpha in vascular smooth muscle cells. Biochem. Pharmacol..

[29] Suzuki M, Kon K, Ikejima K, Arai K, Uchiyama A, Aoyama T (2019). The chemical chaperone 4-phenylbutyric acid prevents alcohol-induced liver injury in obese KK-Ay mice. Alcohol Clin. Exp. Res..

[30] Yue S, Zhu J, Zhang M, Li CH, Zhou XL, Michael M (2016). The myeloid heat shock transcription factor 1/beta-catenin axis regulates NLR family, pyrin domain-containing 3 inflammasome activation in mouse liver ischemia/ reperfusion injury. Hepatology.

[31] Lu L, Yue S, Jiang L, Li C, Zhu Q, Ke M (2018). Myeloid Notch1 deficiency activates the RhoA/ROCK pathway and aggravates hepatocellular damage in mouse ischemic livers. Hepatology.

[32] Neuman MG, Cameron RG, Haber JA, Katz GG, Malkiewicz IM, Shear NH (1999). Inducers of cytochrome P450 2E1 enhance methotrexate-induced hepatocytoxicity. Clin. Biochem..

[33] Oral O, Akkoc Y, Bayraktar O, Gozuacik D (2014). Physiological and pathological significance of the molecular cross-talk between autophagy and apoptosis. Histol. Histopathol..

[34] Naidoo N (2009). Cellular Stress/the unfolded protein response: Relevance to sleep and sleep disorders. Sleep Med. Rev..

