# Supplementary material for: Social defeat stress induces liver injury by modulating endoplasmic reticulum stress in C57BL/6J mice
Source: Sci Rep. 2024 Mar 26;14:7137. doi: 10.1038/s41598-024-57270-0 (PMC10966005; doi:10.1038/s41598-024-57270-0)
Supplement: Supplementary file 1 — Supplementary Information. [file 41598_2024_57270_MOESM1_ESM.pdf]

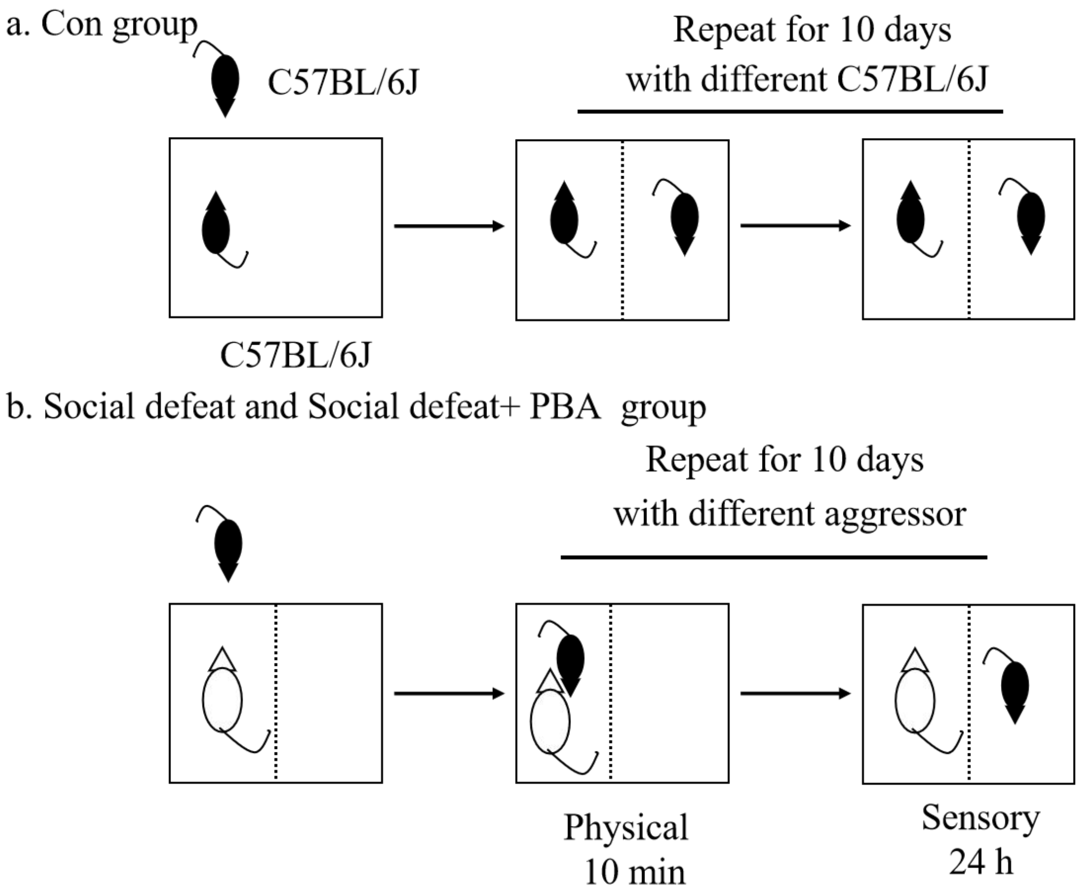

Supplement Figure 1. Experimental design of the social defeat stress paradigm. (a) Control mice were housed in equivalent cages with a plastic perforated divider with the same strain members in a shared home cage. (b) Social defeated mice were exposed to physical interaction with an ICR mouse (aggressor) for 10 minutes and physically defeated; then, they were housed in a shared home cage that was separated by a perforated divider and subjected to psychological stress for 24 hours.

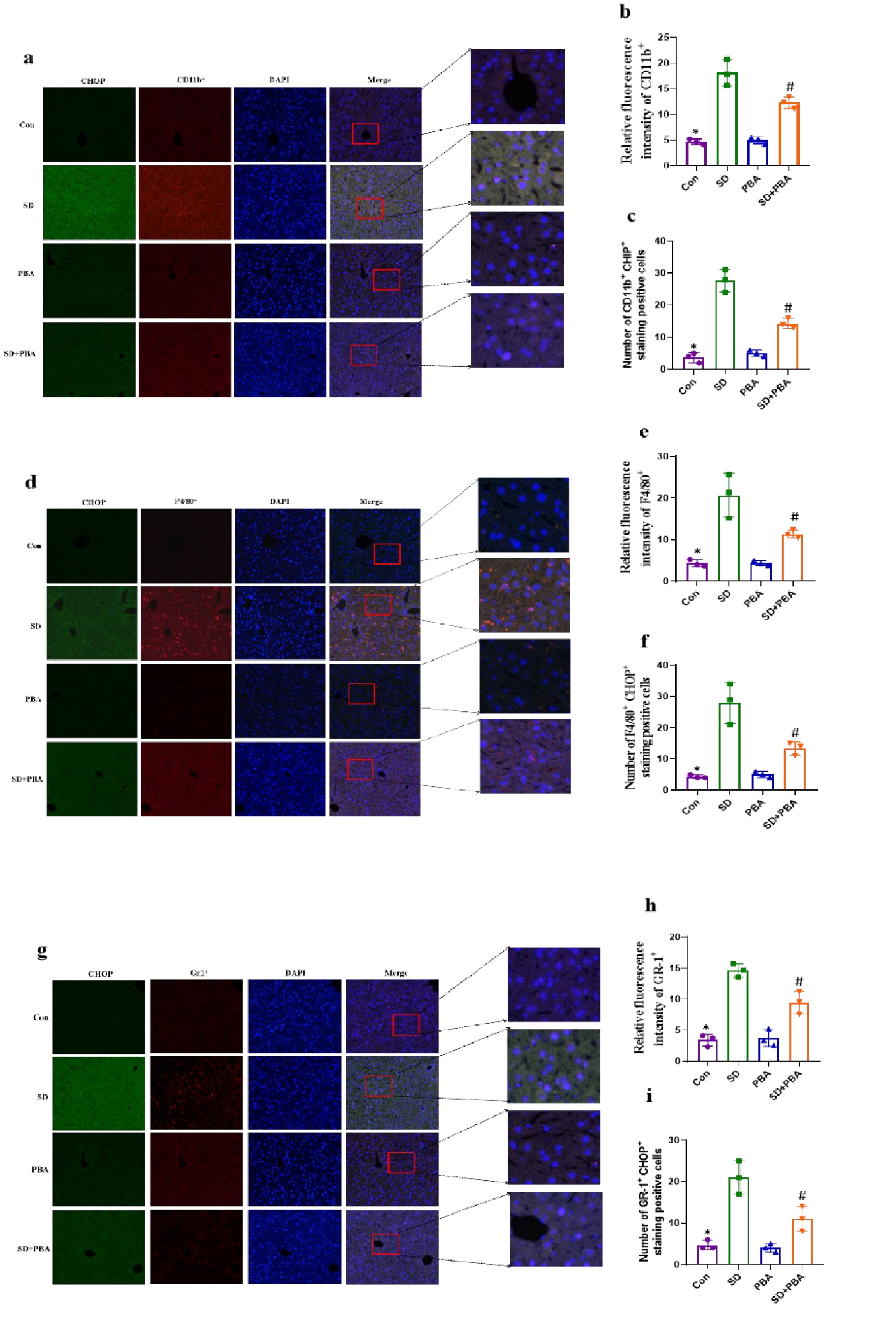

Supplement figure 2. Effects of PBA on ER stress in macrophage induced by social defeat stress. (a) Immunofluorescent staining of CD11b^{+} macrophages, F4/80^{+}

macrophages, Gr-1þ$^{+}$ neutrophils and CHOP in the liver were shown (×100). Relative fluorescence intensity of CD11b^{+} macrophages (b), F4/80^{+} macrophages (e), and Gr-1^{+} neutrophils (i) were determined. Number of CHOP and CD11b^{+} positive cells (c). Number of CHOP and F4/80^{+} positive cells (f). Number of CHOP and Gr-1^{+} positive cells (j). *$p<0.01$ vs con group; #$p<0.01$ vs social defeat stress group. PBA: 4-phenylbutyric acid.

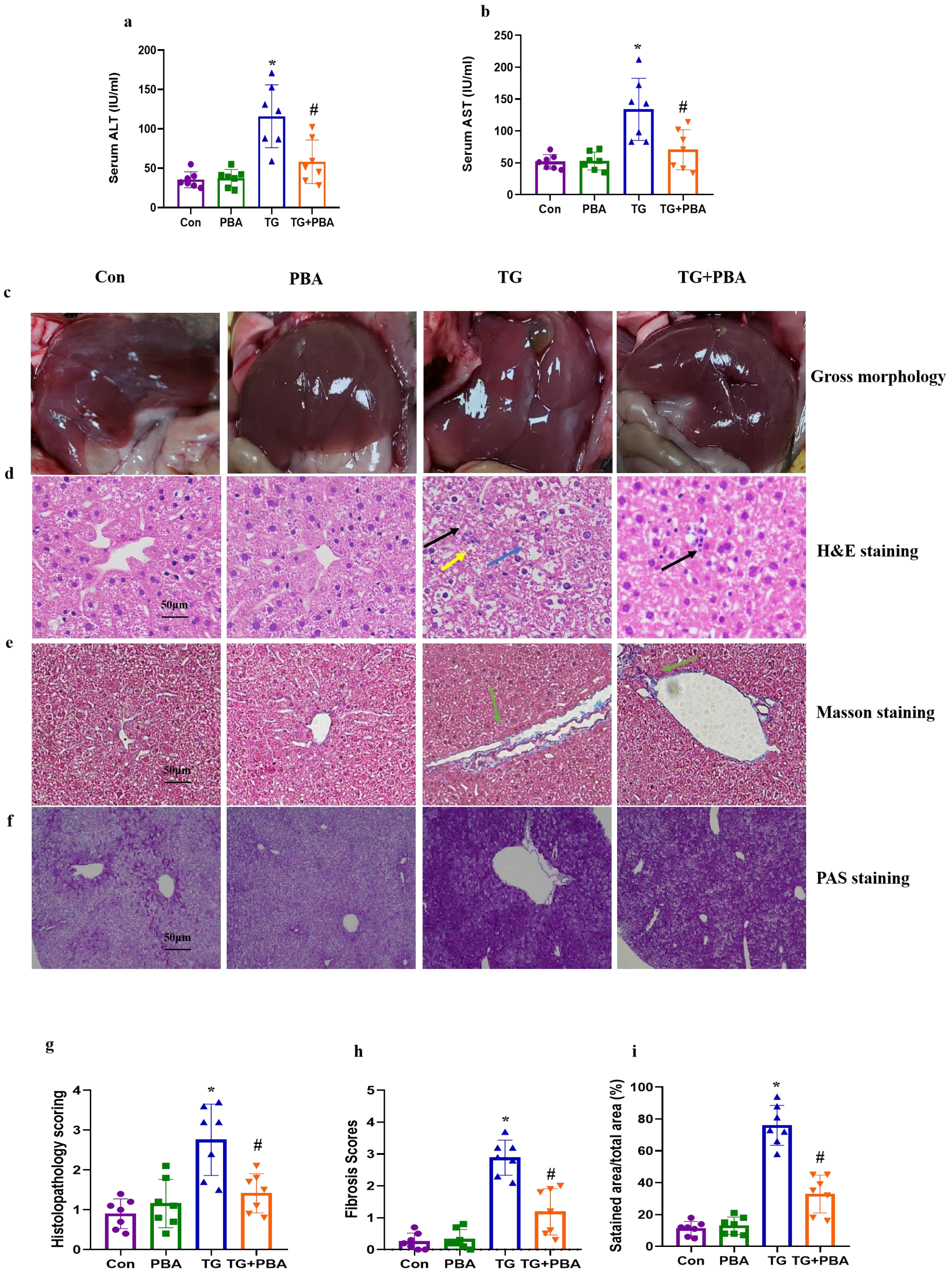

Supplement figure 3. PBA on TG-induced AST, ALT activity and liver injury. Activity of ALT (a) and AST (b) in serum. (c) Gross morphology of liver. (d) H&E staining (×200), (e) Masson staining (×100) and (f) PAS staining (×100). Representative images of each group are presented. (g) Histopathological mean liver injury scores in (c). (h) Quantification for fibrotic areas by Masson staining in (d). (i) Quantification of PAS-stained area in (e). Black arrows indicate inflammatory cell infiltration, Blue arrows indicate balloon degeneration, Yellow arrows indicate

haemorrhage, and Green arrows indicate fibrotic in liver tissue. Data are expressed as mean ± standard deviation. N=7 per group; *p<0.01 vs con group; #p<0.01 vs TG group. PBA: 4-phenylbutyric acid. TG: Thapsigargin.
